# Systematic assessment of HER2/*neu* in gynecologic neoplasms, an institutional experience

**DOI:** 10.1186/s13000-016-0553-8

**Published:** 2016-10-22

**Authors:** Jennifer S. Woo, Sophia K. Apple, Peggy S. Sullivan, Jian-Yu Rao, Nora Ostrzega, Neda A. Moatamed

**Affiliations:** 1 Department of Pathology and Laboratory Medicine, David Geffen School of Medicine at UCLA, 10833 Le Conte Avenue, BOX 951732, 1P-241 CHS, Los Angeles, CA 90095-1732 USA; 2 Department of Pathology and Laboratory Science, Olive View Medical Center, Sylmar, CA 91342 USA

**Keywords:** Endometrium, Ovary, Müllerian, HER2/neu, Immunohistochemistry, FISH

## Abstract

**Background:**

HER2/*neu* overexpression and/or amplification has been widely studied in a number of solid tumors, primarily in the breast. In gynecologic neoplasms, determination of HER2/neu status has not been well studied as a predictive biomarker in anti-HER2/neu treatment.

**Methods:**

We systematically evaluated the HER2/neu reactions by immunohistochemistry and fluorescent in situ hybridization in malignant gynecologic neoplasms as experienced in our institution.

**Results:**

The HER2/neu overexpression or amplification occurred in 8 % of the cancers of the gynecological organs in our series. Majority of the HER2/neu overexpression and/or amplification occurred in clear cell (27 %) and serous (11 %) carcinomas. HER2/neu positivity was also seen in undifferentiated as well as in mixed clear cell and serous carcinomas. Discordant IHC and FISH results (positive by FISH but not IHC) was seen in 2 cases. Majority of the HER2/neu overexpression and/or amplification occurs in the endometrium rather than the ovary. Heterogeneity of the HER2/neu by IHC staining was in < 2 % of the tumors in our series.

**Conclusions:**

We recommend the HER2/neu studies on Müllerian carcinomas of clear cell, serous, and undifferentiated types, particularly when they arise in the endometrium. Since there are some discordant IHC/FISH results, we also propose performing the HER2/neu testing by FISH when the IHC score is less than 3 + .

This work was presented as a poster at the annual meeting of the United States and Canadian Academy of Pathology (USCAP) in Boston Massachusetts in 2015 [1].

## Background

Certain gynecologic cancers such as high grade endometrial and ovarian cancers are a leading cause of morbidity and mortality. Current treatment protocols for both endometrial and ovarian based tumors are largely organ specific and are not defined by histologic subtypes. Certain histologic categories, however, have been associated with poor clinical outcomes. In the endometrium (uterine corpus), serous and clear cell histologic subtypes follow an aggressive clinical course as a result of their high recurrence rate and relative resistance to conventional chemotherapy [2]. In the ovary, clear cell ovarian carcinoma has shown dismal response rates to the therapeutic agents [3]. In treatment of these tumors, targeting the molecular pathway would be the next logical approach when the traditional chemotherapies fail or there is a poor response.

The HER2/neu overexpression/amplification has been widely studied in a number of solid tumors. When overexpressed and/or amplified, there is a well-established targeted therapy when the cancers occur in the breast, esophagus, and stomach [4–6]. Overexpression of HER2/neu has been previously reported in endometrial serous, ovarian mucinous, and ovarian clear cell carcinomas [7–11]. Although the HER2/neu status in female reproductive cancers has been investigated over the past two decades, determination of the HER2/neu status has not been well studied as a predictive biomarker for response to anti-HER2/neu treatment in the gynecologic cancers unlike in the breast and the digestive system. In this study, we systematically evaluated the HER2/neu status of the malignant gynecologic neoplasms, within our institution, which can be used for its effectiveness in the anti-HER2/neu therapies.

## Methods

For the conduct of this study, an approval was obtained from the Institutional Review Board at the David Geffen School of Medicine at UCLA. The surgically excised specimens which had been diagnosed with gynecologic cancers (including endometrial, ovarian, and primary peritoneal tumors) and had either immunohistochemical (IHC) and/or fluorescence in-situ hybridization (FISH) studies for the HER2/neu were selected for this study. These cases were identified by a computer search of the surgical pathology database (2005 to 2014) in our institution. Hereupon, “HER2” is used for reference to HER2/neu gene. Routinely, the HER2 tests by IHC and/or FISH are not performed on all gynecological neoplasms in our medical center. The tests are performed at the request of the clinicians or determination by the pathologists only on the resected tumors. Retrospective demographic and clinical data were collected from a detailed review of medical records, including operative notes and pathology reports. For all neoplastic lesions, when applicable, the FIGO (International Federation of Gynecology and Obstetrics) and nuclear grades were recorded according to the established criteria [12].

Appropriate samples from the surgically excised specimens were selected and fixed in 10 % neutral-buffered formalin for at least 6 h and embedded in paraffin blocks. Four-μm thick sections from the blocks were stained with hematoxylin and eosin (H&E) according to the established laboratory protocol. The HER2-IHC was performed on the duplicate sections of the tissues using the FDA-approved HercepTest™ kit (DAKO, Carpentaria, CA, USA) containing appropriate positive and negative controls [13]. The HER2-FISH assay was performed on the tumor tissue sections using the FDA-approved PathVysion™ HER2 DNA Probe Kit (Abbott Molecular Inc., Des Plaines, IL) [14]. In all cases, at least an area of 1 cm^2^ or greater of the tumor tissue was analyzed for assessing the HER2 reactions.

### HER2 by IHC

Scores were assigned per the current American Society of Clinical Oncology (ASCO)/College of American Pathologists (CAP) guidelines for breast cancer [15]. The HER2 IHC-slides were re-reviewed by the two pathologists (JW and NAM) and scored independently according to the following algorithm [16, 17].

Score 0. No staining or incomplete membrane staining that was faint/barely perceptible in ≤ 10 % of tumor cells, indicating *no overexpression*.
Score 1+. Incomplete membrane staining that was faint/barely perceptible in > 10 % of tumor cells, indicating *no overexpression*.
Score 2+. Complete circumferential moderately intense membranous staining seen in > 10 % of the tumor cells, indicating *equivocal overexpression*.
Score 3+. Circumferential membrane staining that was complete and intense involving > 10 % of tumor cells, indicating *overexpression* of the HER2 gene.

### HER2 by FISH

In the assay, a ratio of HER2 to CEP17 (chromosome enumerating probe-17) signals in 20 cells was used for scoring the FISH test results. A HER2/CEP17 ratio of ≥ 2 was considered as *positive* for amplification of the HER2 gene. If the ratio was < 2, but the signals for HER2 copy number per cell were ≥ 6, also the result was considered *positive* per recommendations by the American Society of Clinical Oncologists and the College of American Pathologists [15, 18]. These recommendations, however, have been for carcinomas of the breast and no such recommendations are made for the HER2 FISH in the cancers of other organs. Therefore, only the HER2/CEP17 ratios of ≥ 2 are used to indicate a positive result for non-breast cancers in our institution as used by others [19, 20].

### Study design

Cases with 3+ HER2 staining pattern of over expression by IHC and/or the HER2/CEP17 ratio of ≥ 2 by FISH were considered as *positive* for HER2 which are indicated as such in the text and the tables. Cases with IHC scores of 0 and 1+ and/or the HER2 ratios of < 2 by FISH were classified as *negative* for HER2. An equivocal IHC staining pattern of 2+ without a corresponding FISH study was excluded from this study. The concordance rate between the IHC and the FISH test results was also recorded.

All the subjects were divided into 5 groups bases on the histological malignancy types; *Group I*, clear cell carcinoma; *Group II*, serous carcinoma; *Group III*, endometrioid adenocarcinoma; *Group IV*, mixed surface epithelial adenocarcinomas; and *Group V*, other malignant neoplasms. For each group, pertinent findings were tabulated including the HER2 reactions. In addition, each case was evaluated for intratumoral heterogeneity of the HER2 reaction by IHC staining as observed by Buza and Hui [21]. The heterogeneity was defined as the presence of two-degree or more difference in staining scores involving at least 5 % of the tumor cells [21].

## Results

During the approximately 9-years period, a total of 125 gynecologic cases were identified per the inclusion criteria which subsequently were evaluated for overexpression of the HER2 by IHC and/or amplification by FISH. All cases had the IHC staining except for one (see Group V below). Only 44 of the 125 patients had the corresponding FISH results. Overall, 8 % (10/125) of the gynecologic neoplasms had the HER2 overexpression/amplification in this series. Discordant IHC and FISH results (positive by FISH but not by IHC) was seen in 20 % (2 of 10) of the HER2 positive cases.

### Group I

**Fig. 1:**
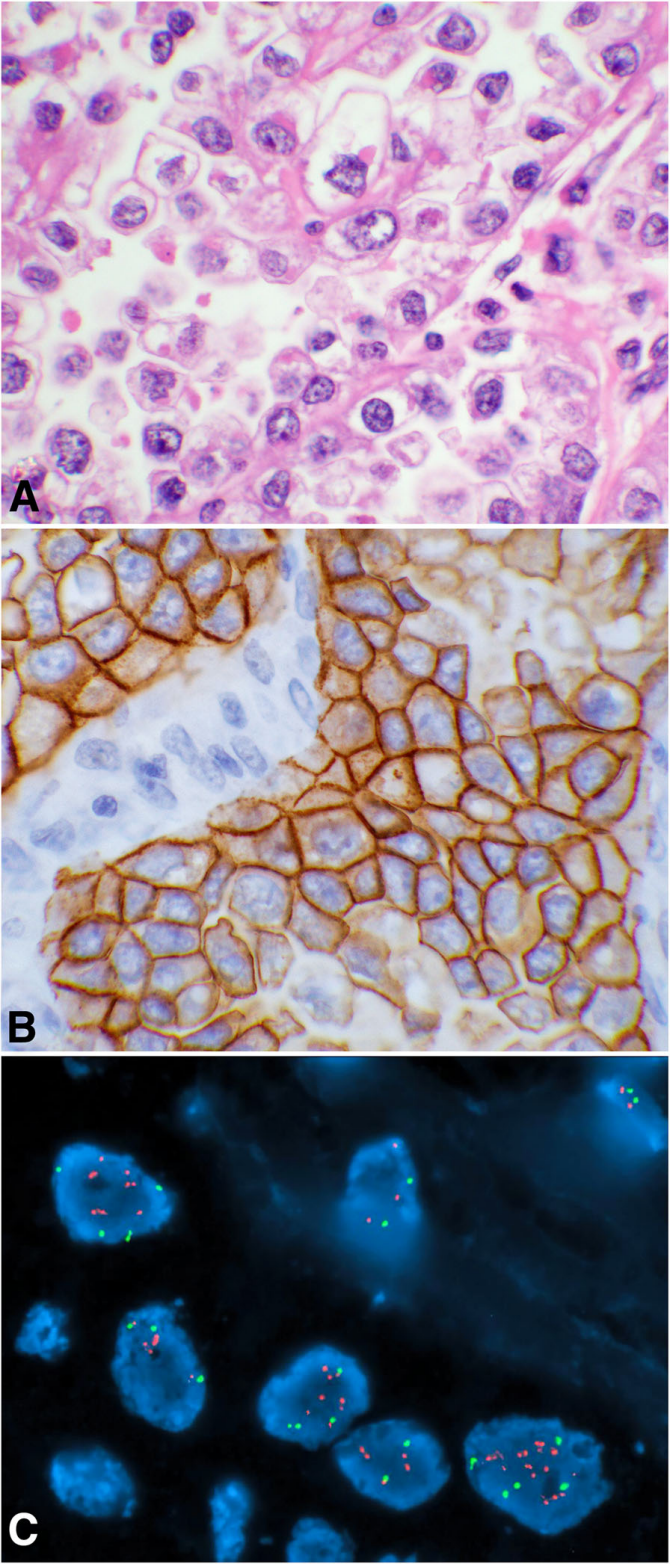
An example of a clear cell carcinoma, with the HER2 overexpression by IHC and amplification by FISH, is shown (case # 1; Table 1). Panel (**a**) shows a photomicrograph of hematoxylin and eosin stain (40× objective) of the tumor showing characteristic nuclear and cytoplasmic morphology. The HER2 overexpression is displayed showing a strong membrane staining by IHC (panel **b**, 40× objective) and the HER2 gene (*red signals*) amplification by FISH (panel **c**)

This group was comprised of cases with a histological diagnosis of clear cell carcinoma. There were 11 subjects in this group (Table 1). Patients’ ages ranged from 37 to 89 with a median of 54 years old. Three (27.3 %) of these cases were positive for HER2. Two of which had corresponding and concordant FISH, while one had only overexpression by IHC and FISH had not been performed (case # 3; Table 1). One of the three positive HER2 cases was in the ovary and the other two were in the endometrium which constituted 9.1 % and 18.2 % in this group respectively. An example of a case with overexpression of the HER2 by IHC and amplification by FISH (case # 1; Table 1) is shown in Fig. 1.

**Table 1:** Group I, cases diagnosed with *clear cell carcinomas*

Case no.	HER2/neu			Primary site
	IHC	FISH	Result	
**1**	**3+**	**2.58**	**POS**	**Ovary**
**2**	**2+**	**2.89**	**POS**	**Endometrium**
**3**	**3+**	**NP**	**POS**	**Endometrium**
4	0	1.1	NEG	Ovary
5	0	NP	NEG	Ovary
6	0	NP	NEG	Ovary
7	1+	NP	NEG	Ovary
8	0	NP	NEG	Endometrium
9	1+	NP	NEG	Endometrium
10	1+	NP	NEG	Endometrium
11	1+	NP	NEG	Endometrium

Bold data signify the positive results

*IHC* immunohistochemistry score, *FISH* fluorescence in-situ hybridization HER2/CEP17 ratio, *NP* not performed, *POS* positive, *NEG* negative

### Group II

**Fig. 2:**
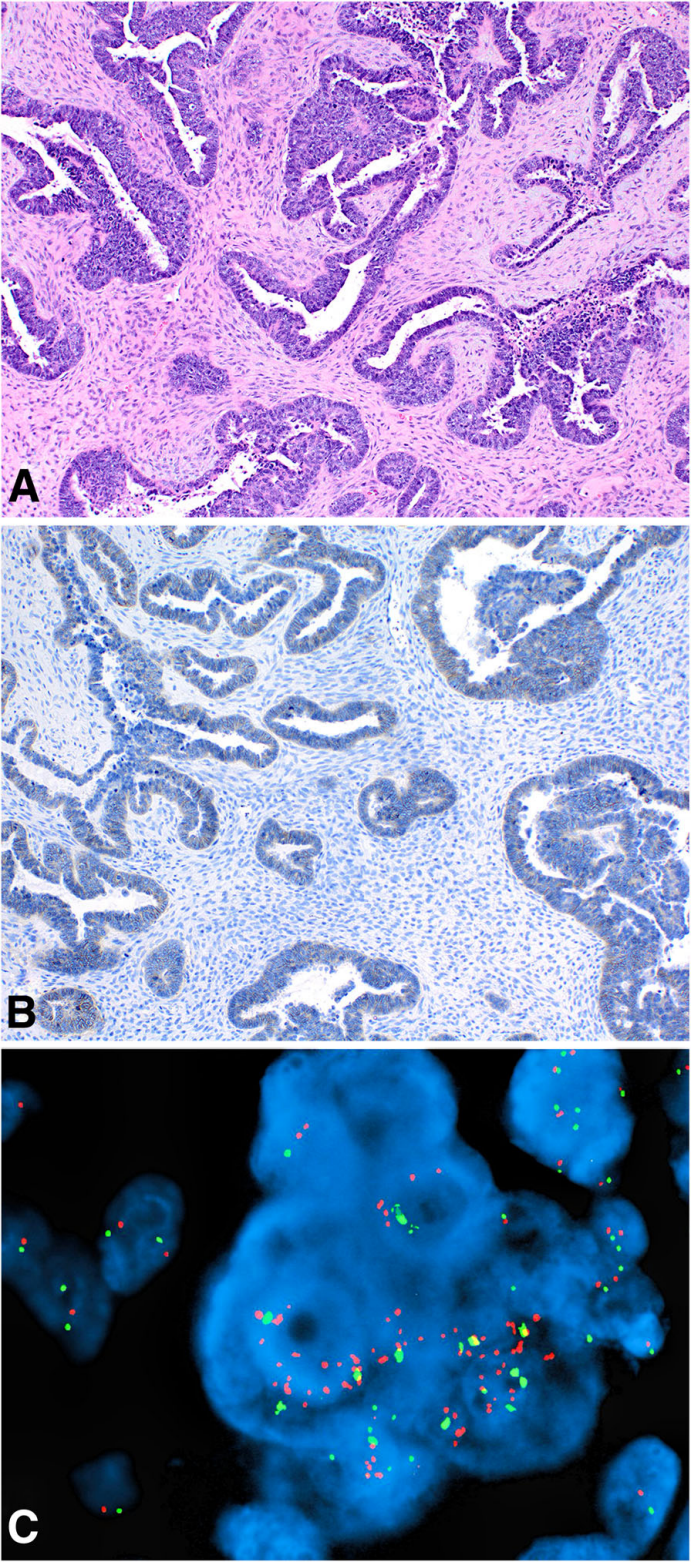
An example of serous carcinoma, with discordant IHC expression and FISH amplification (case # 1; Table 2), is shown. Note the hematoxylin and eosin stain (panel **a**, 10× objective), negative HER2 expression by IHC (panel **b**, 10× objective), and the HER2 gene (*red signals*) amplification by FISH (panel **c**)

This group was comprised of cases with a histological diagnosis of serous carcinoma. There were 45 subjects in this group (Table 2). Patients’ ages ranged from 32 to 83 with a median of 58 years old. Forty-four cases were of high grade and only one case had a low grade serous carcinoma (case # 11; Table 2). All cases in this group had the HER2 tested by IHC while 16 of them were accompanied by the FISH assay. Five (11.1 %) of these cases were positive for the HER2, 2 by FISH (cases # 1–2; Table 2), 2 by IHC and FISH (cases # 3–4; Table 2), and 1 by IHC (case # 5; Table 2). The first two cases had a score of 0 by IHC (cases # 1 and 2; Table 2) indicating discordancy. In this Group, the positive HER2 cases were 1 (2.2 %) in the ovary and 4 (8.9 %) in the endometrium (Table 2). An example of a discordant case of ovarian serous carcinoma (case # 1; Table 2) is shown in Fig. 2. Four (# 3, 35, 44, & 45; Table 2) of the 16 cases with corresponding FISH results had an IHC score of 2+. Of which only one showed the HER2 amplification by FISH (case # 3; Table 2) while the other 3 did not (cases # 35, 44 & 45; Table 2).

**Table 2:** Group II, cases diagnosed with *serous carcinoma*

Case no.	HER2 amplification			Primary site	Histopathology diagnosis		
	IHC	FISH	Result		Type	FIGO	Nuc
**1**	**0**	**2.75**	**POS**	**Ovary**	**SCA**	**3**	**3**
**2**	**0**	**2.26**	**POS**	**Endometrium**	**SCA**	**3**	**3**
**3**	**2+**	**5.16**	**POS**	**Endometrium**	**SCA**	**3**	**3**
**4**	**3+**	**6.1**	**POS**	**Endometrium**	**SCA**	**3**	**3**
**5**	**3+**	**NP**	**POS**	**Endometrium**	**SCA**	**3**	**3**
6	0	1	NEG	Fallopian Tube	SCA	3	3
7	0	1	NEG	Ovary	SCA	3	3
8	0	1.03	NEG	Ovary	SCA	3	3
9	0	1.06	NEG	Ovary	SCA	3	3
10	0	1.4	NEG	Ovary	SCA	3	3
11	0	NP	NEG	Ovary	SCA	1	1
12	0	NP	NEG	Ovary	SCA	3	3
13	0	NP	NEG	Ovary	SCA	3	3
14	0	NP	NEG	Ovary	SCA	3	3
15	0	NP	NEG	Ovary	SCA	3	3
16	0	NP	NEG	Ovary	SCA	3	3
17	0	NP	NEG	Ovary	SCA	3	3
18	0	NP	NEG	Ovary	SCA	3	3
19	0	NP	NEG	Ovary	SCA	3	3
20	0	NP	NEG	Ovary	SCA	3	3
21	0	NP	NEG	Ovary	SCA	3	3
22	1+	1	NEG	Ovary	SCA	3	3
23	1+	1	NEG	Ovary	SCA	3	3
24	1+	1.2	NEG	Ovary	SCA	3	3
25	1+	NP	NEG	Ovary	SCA	3	3
26	1+	NP	NEG	Ovary	SCA	3	3
27	1+	NP	NEG	Ovary	SCA	3	3
28	1+	NP	NEG	Ovary	SCA	3	3
29	1+	NP	NEG	Ovary	SCA	3	3
30	1+	NP	NEG	Ovary	SCA	3	3
31	1+	NP	NEG	Ovary	SCA	3	3
32	1+	NP	NEG	Ovary	SCA	3	3
33	1+	NP	NEG	Ovary	SCA	3	3
34	1+	NP	NEG	Ovary	SCA	3	3
35	2+	1.62	NEG	Ovary	SCA	3	3
36	0	1.08	NEG	Peritoneum	SCA	3	3
37	0	NP	NEG	Peritoneum	SCA	3	3
38	0	NP	NEG	Peritoneum	SCA	3	3
39	1+	NP	NEG	Peritoneum	SCA	3	3
40	1+	NP	NEG	Peritoneum	SCA	3	3
41	1+	NP	NEG	Endometrium	SCA	3	3
42	1+	NP	NEG	Endometrium	SCA	3	3
43	1+	NP	NEG	Endometrium	SCA	3	3
44	2+	0.91	NEG	Endometrium	SCA	3	3
45	2+	1.21	NEG	Endometrium	SCA	3	3

Bold data signify the positive results

*IHC* immunohistochemistry score, *FISH* fluorescence in-situ hybridization HER2/CEP17 ratio, *FIGO* FIGO grade, *Nuc* nuclear grade, *NP* not performed, *POS* positive, *NEG* negative, *SCA* serous carcinoma

### Group III

This group was comprised of cases with a histological diagnosis of endometrioid adenocarcinoma. There were 28 subjects in this group. Patients’ ages ranged from 31 to 86 with a median of 61.5 years old with varied FIGO and nuclear grading from 1 to 3. All cases in this group had the HER2 test by IHC while 6 of them were accompanied by the FISH assay. None (0.0 %) of these cases were positive for the HER2 (Table 3).

**Table 3:** Group III, cases diagnosed with *endometrioid adenocarcinomas*

Case no.	HER2/neu			Primary site	Histopathology diagnosis		
	IHC	FISH	Result		Type	FIGO	Nuc
1	0	1.4	NEG	Endometrium	ENAdCA	2	3
2	0	1.15	NEG	Endometrium	ENAdCA	2	2
3	0	1.05	NEG	Endometrium	ENAdCA	2	2
4	1+	0.98	NEG	Endometrium	ENAdCA	1	1
5	1+	0.98	NEG	Endometrium	ENAdCA	1	2
6	0	NP	NEG	Fallopian Tube	ENAdCA	2	2
7	1+	NP	NEG	Endometrium	ENAdCA	1	2
8	1+	1	NEG	Endometrium	ENAdCA	1	1
9	1+	NP	NEG	Endometrium	ENAdCA	2	2
10	1+	NP	NEG	Ovary	ENAdCA	3	3
11	0	NP	NEG	Endometrium	ENAdCA	1	1
12	1+	NP	NEG	Endometrium	ENAdCA	2	3
13	0	NP	NEG	Endometrium	ENAdCA	2	2
14	0	NP	NEG	Ovary	ENAdCA	3	3
15	1+	NP	NEG	Endometrium	ENAdCA	2	2
16	1+	NP	NEG	Endometrium	ENAdCA	2	2
17	1+	NP	NEG	Endometrium	ENAdCA	3	2
18	0	NP	NEG	Ovary	ENAdCA	3	3
19	1+	NP	NEG	Endometrium	ENAdCA	1	2
20	0	NP	NEG	Endometrium	ENAdCA	2	1
21	1+	NP	NEG	Endometrium	ENAdCA	1	2
22	1+	NP	NEG	Endometrium	ENAdCA	1	2
23	1+	NP	NEG	Endometrium	ENAdCA	1	1
24	1+	NP	NEG	Endometrium	ENAdCA	2	1
25	0	NP	NEG	Ovary	ENAdCA	3	3
26	1+	NP	NEG	Endometrium	ENAdCA	2	1
27	0	NP	NEG	Endometrium	ENAdCA	2	2
28	0	NP	NEG	Ovary	ENAdCA	3	3

*IHC* immunohistochemistry score, *FISH* fluorescence in-situ hybridization HER2/CEP17 ratio, *FIGO* FIGO grade, *Nuc* nuclear grade, *NP* not performed, *NEG* negative, *ENAdCA* endometrioid adenocarcinoma

### Group IV

**Fig. 3:**
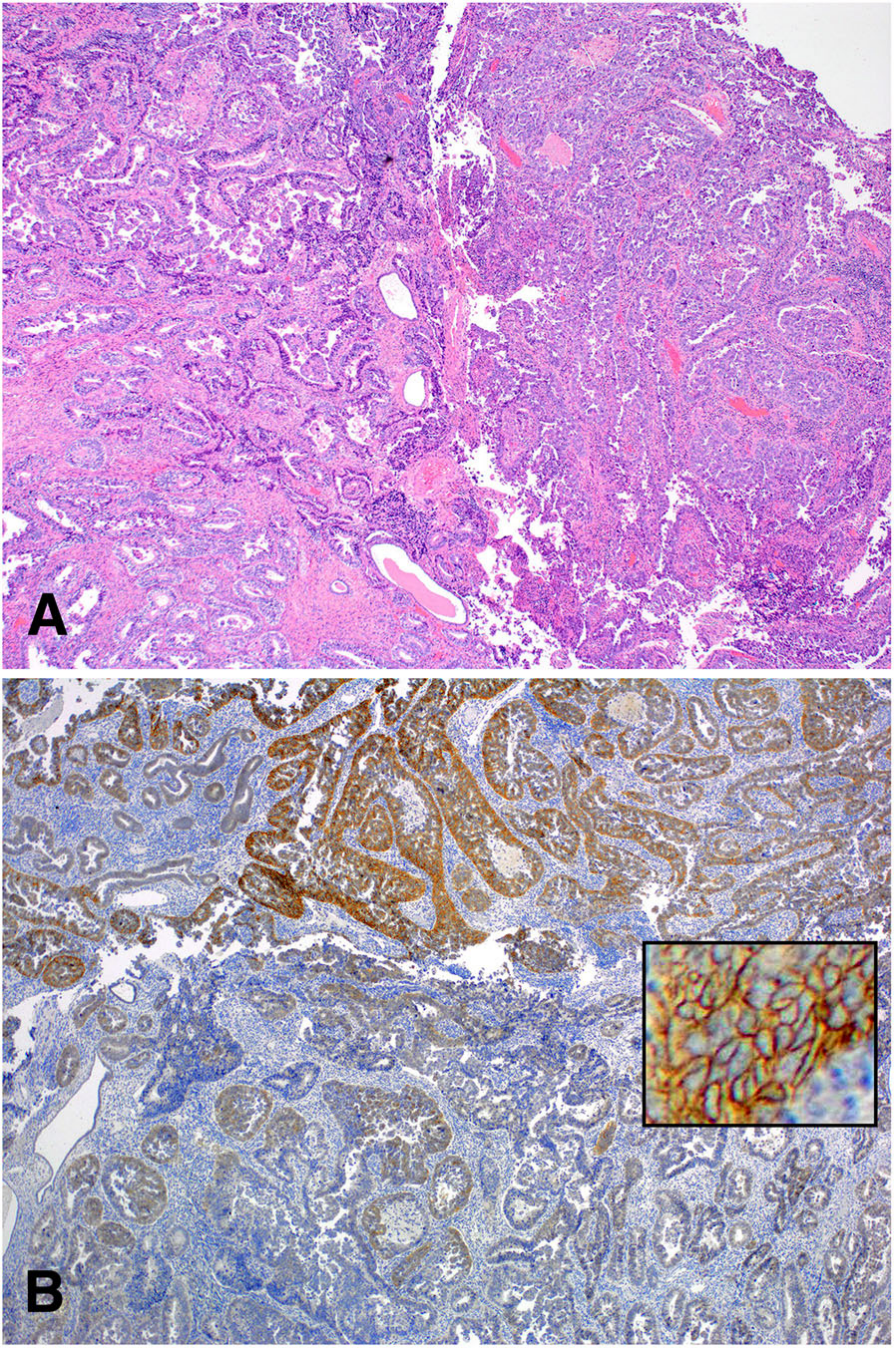
An example of a case with intratumoral HER2 heterogeneity (case # 20; Table 4) is shown with its hematoxylin and eosin stain counterpart (panel **a**, 10× objective). The HER2 by IHC shows a strong reaction in the upper and a weak or no reaction in the lower parts of the photomicrograph (panel **b**, 10× objective). The inset in panel B shows strong cell membrane staining of the HER2 by IHC in the overexpressed part of the tumor (40× objective)

This group was comprised of cases with the histological diagnosis of mixed surface epithelial adenocarcinomas. The mixed surface epithelial carcinomas included clear cell, serous, endometrioid, and mucinous types. There were 26 subjects in this group. Patients’ ages ranged from 30 to 78 with a median of 64 years old. Only one (3.8 %) case, in this Group, was positive for the HER2 amplification with concordant IHC positivity which had occurred in the endometrium (Case #1; Table 4). This case had a histologic type of mixed clear cell and serous carcinomas. Two (7.7 %) of the cases (cases # 7 & 20; Table 4) in this group had intratumoral heterogeneity as described above [21]. The patterns of the IHC staining ranged from 0 to 3+ in both cases, where the 3+ reaction was seen in less than 10 % of the tumor cells. One of the examples of the heterogeneity of the reaction (case # 20; Table 4) is shown in Fig. 3. Overall, these two cases represented 1.6 % (2 of 125) heterogeneity in all five groups.

**Table 4:** Group IV, cases diagnosed with *mixed surface epithelial carcinomas*

Case no.	HER2/neu			Primary site	Histopathology diagnosis		
	IHC	FISH	Result		Type	FIGO	Nuc
**1**	**2+**	**7.41**	**POS**	**Endometrium**	**CCCA + SCA**	**3**	**3**
2	1+	1.02	NEG	Ovary	CCCA + SCA	3	3
3	0	1.37	NEG	Pelvic mass	CCCA + SCA	3	3
4	0	NP	NEG	Ovary	CCCA + SCA	3	3
5	0	NP	NEG	Ovary	CCCA + SCA	3	3
6	0	NP	NEG	Ovary	CCCA + SCA	3	3
7^a^	0–3+	1.38	NEG	Endometrium	CCCA + SCA	3	3
8	1+	0.93	NEG	Endometrium	CCCA + ENAdCA	3	3
9	1+	NP	NEG	Endometrium	CCCA + ENAdCA	3	3
10	1+	NP	NEG	Endometrium	CCCA + ENAdCA	3	3
11	0	NP	NEG	Ovary	CCCA + ENAdCA	3	3
12	1+	NP	NEG	Ovary	CCCA + ENAdCA	3	3
13	0	NP	NEG	Ovary	CCCA + ENAdCA	3	3
14	0	NP	NEG	Endometrium	SCA + ENAdCA	3	3
15	1+	NP	NEG	Ovary	SCA + ENAdCA	3	3
16	0	NP	NEG	Peritoneum	SCA + ENAdCA	3	3
17	0	NP	NEG	Endometrium	SCA + ENAdCA	3	3
18	0	NP	NEG	Ovary	SCA + ENAdCA	3	3
19	1+	1.7	NEG	Endometrium	SCA + ENAdCA	3	3
20^a^	0–3+	1.14	NEG	Endometrium	SCA + ENAdCA	3	3
21	0	NP	NEG	Ovary	SCA + ENAdCA	3	3
22	1+	1.2	NEG	Endometrium	SCA + ENAdCA	3	3
23	0	1.04	NEG	Ovary	SCA + ENAdCA	3	3
24	1+	1	NEG	Ovary	SCA + ENAdCA	3	3
25	1+	1	NEG	Ovary	SCA + ENAdCA + Mu	3	3
26	0	1	NEG	Endometrium	ENAdCA + Mu	1	1

Bold data signify the positive results

*IHC* immunohistochemistry score, *FISH* fluorescence in-situ hybridization HER2/CEP17 ratio, *FIGO* FIGO grade, *Nuc* nuclear grade, *NP* not performed, *POS* positive, *NEG* negative, *CCCA* clear cell carcinoma, *SCA* serous carcinoma, *ENAdCA* endometrioid adenocarcinoma, *Mu* mucinous adenocarcinoma

^a^ Denotes intratumoral heterogeneity of HER2 reaction

### Group V

This group was comprised of other neoplasms including cases with histological diagnoses of undifferentiated carcinoma of the endometrium, endocervical adenocarcinoma, endometrial stromal sarcoma (ESS), malignant mixed müllerian tumor (MMMT), and yolk sac tumor of the ovary. There were 15 subjects in this group. Patients’ ages ranged from 20 to 72 with a median of 54 years old. Except for one (6.7 %), remaining 14 cases were negative for the HER2 (Table 5). Of the 4 patients with a diagnosis of undifferentiated carcinoma (UnDiff), one had the HER2 amplification (case #1; Table 5) with a concordant IHC test.

**Table 5:** Group V, cases diagnosed with *other malignant neoplasms*

Case no.	HER2/neu			Primary site	Histopathology diagnosis		
	IHC	FISH	Result		Type	FIGO	Nuc
**1**	**3+**	**3.5**	**POS**	**Endometrium**	**UnDiff**	**3**	**3**
2	0	NP	NEG	Endometrium	UnDiff	3	3
3	1+	NP	NEG	Endometrium	UnDiff	3	3
4	NP	1	NEG	Endometrium	UnDiff	3	3
5	0	NP	NEG	Ovary	MMMT	NA	NA
6	0	1.54	NEG	Endometrium	MMMT	NA	NA
7	0	NP	NEG	Endometrium	MMMT	NA	NA
8	1+	NP	NEG	Endometrium	MMMT	NA	NA
9	1+	NP	NEG	Pelvic Mass	MMMT	NA	NA
10	1+	1	NEG	Endometrium	MMMT	NA	NA
11	1+	NP	NEG	Ovary	MMMT	NA	NA
12	0	NP	NEG	Endometrium	ESS	NA	NA
13	0	NP	NEG	Endometrium	ESS	NA	NA
14	1+	NP	NEG	Cervix	ECAdCA	1	1
15	0	NP	NEG	Ovary	Yolk Sac Tumor	NA	NA

Bold data signify the positive results

*IHC* immunohistochemistry score, *FISH* fluorescence in-situ hybridization HER2/CEP17 ratio, *FIGO* FIGO grade, *Nuc* nuclear grade, *NP* not performed, *POS* positive, *NEG* negative, *UnDiff* undifferentiated carcinoma, *ECAdCA* endocervical adenocarcinoma, *MMMT* malignant mixed Müllerian tumor, *ESS* endometrial stromal sarcoma, *NA* not applicable

All cases were further summarized in Table 6 showing the numerical values as well as percentages of the HER2 positivity for each group. Among the cases with the HER2 positivity, clinical follow-ups were available only for 4 patients.

**Table 6:** Summary of overall findings including HER2/neu reactions in the 125 cases

Overall information					HER2/neu	
Groups	Neoplasm	Median age	n	%	POS (n)	POS (%)
Group I	CCCA	54	11	8.8 %	3	27.3 %
Group II	SCA	58	45	36.0 %	5	11.1 %
Group III	ENAdCA	61.5	28	22.4 %	0	0.0 %
Group IV	MxSEAdCA	65	26	20.8 %	1	3.8 %
Group V	Other	54	15	12.0 %	1	6.7 %

*CCCA* clear cell carcinoma, *SCA* serous carcinoma, *ENAdCA* endometrioid adenocarcinoma, *MxSEAdCA* mixed surface epithelial adenocarcinomas, *Other* other neoplasms, *POS* positive

In Table 7, the subjects were rearranged based on the tumor primary sites or anatomical locations in each group. In all five groups, there were 58 neoplasms of the endometrium of which 8 (13.8 %) were HER2 positive. There were 56 ovarian tumors of which 2 (3.6 %) were positive for the HER2. The remaining 11 cases had neoplasms in locations other than endometrium and ovary which had no HER2 positivity (Table 7). The percentages of the positive HER2 cases, in each group, were recalculated for the respective sites of origin. Using this arrangement, serous carcinomas (44.4 %) formed the majority of the HER2 positivity followed by clear cell (33.3 %), mixed epithelial (9.1 %) carcinomas, and other neoplasms (10 %) of the endometrium. The only positive HER2 case in Group V was the one of the four undifferentiated carcinomas (Table 5). In all, 80 % (8 of 10) of the HER2 positive neoplasms were in the endometrium while 20 % positivity had occurred in the ovary. No positive HER2 reactions were observed in other sites (Table 7). In addition, none of the endometrioid adenocarcinomas showed such a positivity.

**Table 7:** Arrangement by the tumor sites in each *Group*

Primary Site	Group I		Group II		Group III		Group IV		Group V	
	CCCA		SCA		ENAdCA		MxSEAdCA		Other	
	n	HER2+	n	HER2+	n	HER2+	n	HER2+	n	HER2+
Endometrium	6	**2 (33.3 %)**	9	**4 (44.4 %)**	22		11	**1 (9.1 %)**	10	**1 (10.0 %)**
Ovary	5	**1 (20.0 %)**	30	**1 (3.3 %)**	5		13		3	
Fallopian Tube			1		1					
Peritoneum			5				1			
Pelvic Mass							1		1	
Cervix									1	
All Sites	11	**3 (27.3 %)**	45	**5 (11.1 %)**	28	0 (0.0 %)	26	**1 (3.9 %)**	15	**1 (6.7 %)**

Bold data signify the positive results

*CCCA* clear cell carcinoma, *SCA* serous carcinoma, *ENAdCA* endometrioid adenocarcinoma, *MxSEAdCA* mixed surface epithelial adenocarcinoma, *Other* other neoplasms, *HER2+* positive HER2/neu

The four cases with the positive HER2 tests were treated with Transtuzumab followed by surgical removal. They responded well initially measured by the imaging studies.

## Discussion

In our experience, the HER2 amplification or overexpression occurs in 8 % of the patients with cancer of the female Müllerian organs. Therefore, it becomes imperative to test the cancer tissues for overexpression of the HER2 for its potential therapeutic impact. When all the anatomical sites are considered, majority of the HER2 overexpression and/or amplification occur in clear cell carcinomas (Tables 1 and 7) at a rate of greater than 27 %. The next tumor type is serous carcinoma which has a rate of 11 % of the HER2 overexpression or amplification (Tables 2 and 7). When stratified by the tumor primary sites (Table 7), serous carcinoma of the endometrium has the highest incidence of the HER2 positivity (44.4 %) which is in agreement with other studies reporting similar findings [19, 21–26]. In general, 80 % of the tumors with the HER2 overexpression/amplification occur in the endometrium and 20 % in the ovary. It appears that undifferentiated carcinomas may also exhibit similar HER2 positivity (Table 5) which is contrary to a published study [27].

Amplification or overexpression of the HER2 in these tumor types, as part of type II endometrial adenocarcinomas, has also been reported by other investigators [8]. Unlike other studies, this series contains all the female Müllerian organs. In this comprehensive systematic evaluation, it becomes clear that the majority of the HER2 amplification occurs in the endometrium rather than the ovary while no such amplification occurs in other organs or sites in this series.

Heterogeneity of the HER2 by IHC staining was seen in less than 2 % of the cases in our series while a higher percentage has been reported by others. Buza et al. have shown that 35 % of the endometrial serous carcinomas had the HER2 overexpression and/or gene amplification in their series where 53 % of which had significant heterogeneity of the protein expression by IHC [19]. They have reported the pattern of the IHC reaction as lack of apical membrane staining resulting in a lateral/basolateral reactive pattern [19].

It has been shown that the prevalence of the HER2 positivity in the Asian female population with ovarian mucinous tumors was 18 % with 100 % concordance rate between IHC and FISH [10]. In our study none of our mucinous tumors showed the HER2 overexpression or amplification.

In endometrial and ovarian carcinomas, as in breast cancer, there have been several studies showing the amplification of the HER2 gene is associated with chemo-resistance and poor prognosis. Transtuzumab combined with chemotherapy agents is currently being investigated in clinical trials in some institutions [28]. In addition to the positive IHC and FISH findings, we have the follow-up for three cases in this study. Two of the three patients had responded well to the treatment which makes transtuzumab a promising therapeutic agent in cases with the HER2 amplification of the Müllerian organs. Future studies should include more comprehensive follow ups in relation to the anti-HER2 targeted therapies. In this series, we only had follow-ups on four patients.

One point of caution is the inclusion criteria for collection of the cases in this series where the selection was based on the HER2 testing if it had been ordered by the clinicians or the pathologists on some cases. Therefore, the results may be somewhat skewed due to the proclivities inherent in the practice of our clinicians and the routines in our institution. Nevertheless, as mentioned earlier, our findings are in agreement with other investigators’ findings in relation to serous carcinomas particularly when tumors were broken down based on their anatomical locations as displayed in Table 7 [19, 21–26]. Despite the bias due to the case selection process, our findings signify the importance of the HER2 testing in particular gynelogical cancers.

## Conclusions

In conclusion, we recommend performing the HER2 tests on gynecologic carcinomas of clear cell, serous, and undifferentiated types particularly when they arise in the endometrium. Since there are some discordant IHC/FISH results, we also propose performing the HER2 testing by both IHC and FISH assays when IHC score is less than 3+ as exhibited well by the cases in this series (Table 2).

## Abbreviations

CCCA: Clear cell carcinoma
CEP: Chromosome enumerating probe
ECAdCA: Endocervical adenocarcinoma
ENAdCA: Endometrioid adenocarcinoma
ESS: Endometrial stromal sarcoma
FDA: Food and Drug Administration
FIGO: International Federation of Gynecology and Obstetrics
FISH: Fluorescence in-situ hybridization
HER2: Human epidermal growth factor receptor 2
IHC: Immunohistochemistry
MMMT: Malignant mixed Müllerian tumor
Mu: Mucinous adenocarcinoma
MxSEAdCA: Mixed surface epithelial adenocarcinomas
NA: Not applicable
NEG: Negative
NP: Not performed
NP: Not performed
Nuc: Nuclear grade
POS: Positive
SCA: Serous carcinoma
UnDiff: Undifferentiated carcinoma

## References

[1] Woo J, Sullivan P, Rao J, Ostrzega N, Apple S, Moatamed N (2015). Assessment of HER2/neu in uterine, ovarian, and peritoneal carcinomas: A retrospective study of 134 cases. Mod Pathol.

[2] Siegel R, Naishadham D, Jemal A (2013). Cancer statistics, 2013. CA Cancer J Clin.

[3] Sugiyama T, Kamura T, Kigawa J (2000). Clinical characteristics of clear cell carcinoma of the ovary: a distinct histologic type with poor prognosis and resistance to platinum-based chemotherapy. Cancer.

[4] Pegram MD, Konecny G, Slamon DJ (2000). The molecular and cellular biology of HER2/neu gene amplification/overexpression and the clinical development of herceptin (trastuzumab) therapy for breast cancer. Cancer Treat Res.

[5] Chan DS, Twine CP, Lewis WG (2012). Systematic review and meta-analysis of the influence of HER2 expression and amplification in operable oesophageal cancer. J Gastrointest Surg.

[6] Hechtman JF, Polydorides AD (2012). HER2/neu gene amplification and protein overexpression in gastric and gastroesophageal junction adenocarcinoma: a review of histopathology, diagnostic testing, and clinical implications. Arch Pathol Lab Med.

[7] Bookman MA, Darcy KM, Clarke-Pearson D, Boothby RA, Horowitz IR (2003). Evaluation of monoclonal humanized anti-HER2 antibody, trastuzumab, in patients with recurrent or refractory ovarian or primary peritoneal carcinoma with overexpression of HER2: a phase II trial of the Gynecologic Oncology Group. J Clin Oncol.

[8] Konecny GE, Santos L, Winterhoff B (2009). HER2 gene amplification and EGFR expression in a large cohort of surgically staged patients with nonendometrioid (type II) endometrial cancer. Br J Cancer.

[9] Lin WL, Kuo WH, Chen FL (2011). Identification of the coexisting HER2 gene amplification and novel mutations in the HER2 protein-overexpressed mucinous epithelial ovarian cancer. Ann Surg Oncol.

[10] Chao WR, Lee MY, Lin WL, Koo CL, Sheu GT, Han CP (2014). Assessing the HER2 status in mucinous epithelial ovarian cancer on the basis of the 2013 ASCO/CAP guideline update. Am J Surg Pathol.

[11] Mentrikoski MJ, Stoler MH (2014). HER2 immunohistochemistry significantly overestimates HER2 amplification in uterine papillary serous carcinomas. Am J Surg Pathol.

[12] Zaino RJ, Kurman RJ, Diana KL, Morrow CP (1995). The utility of the revised International Federation of Gynecology and Obstetrics histologic grading of endometrial adenocarcinoma using a defined nuclear grading system. A Gynecologic Oncology Group study. Cancer.

[13] Dako Corporation (2014). HercepTest™ Interpretation Manual Breast Cancer.

[14] Abbott Molecular Inc (2013). PathVysion Her-2 DNA Probe Kit.

[15] Wolff AC, Hammond ME, Hicks DG (2013). Recommendations for human epidermal growth factor receptor 2 testing in breast cancer: American Society of Clinical Oncology/College of American Pathologists clinical practice guideline update. J Clin Oncol.

[16] Wolff AC, Hammond ME, Schwartz JN (2007). American Society of Clinical Oncology/College of American Pathologists guideline recommendations for human epidermal growth factor receptor 2 testing in breast cancer. Arch Pathol Lab Med.

[17] Wolff AC, Hammond ME, Schwartz JN (2007). American Society of Clinical Oncology/College of American Pathologists guideline recommendations for human epidermal growth factor receptor 2 testing in breast cancer. J Clin Oncol.

[18] Wolff AC, Hammond ME, Hicks DG (2014). Recommendations for human epidermal growth factor receptor 2 testing in breast cancer: American Society of Clinical Oncology/College of American Pathologists clinical practice guideline update. Arch Pathol Lab Med.

[19] Buza N, English DP, Santin AD, Hui P (2013). Toward standard HER2 testing of endometrial serous carcinoma: 4-year experience at a large academic center and recommendations for clinical practice. Mod Pathol.

[20] Growdon WB, Groeneweg J, Byron V (2015). HER2 over-expressing high grade endometrial cancer expresses high levels of p95HER2 variant. Gynecol Oncol.

[21] Buza N, Hui P (2013). Marked heterogeneity of HER2/NEU gene amplification in endometrial serous carcinoma. Genes Chromosomes Cancer.

[22] Santin AD, Bellone S, Gokden M (2002). Overexpression of HER-2/neu in uterine serous papillary cancer. Clin Cancer Res.

[23] Villella JA, Cohen S, Smith DH, Hibshoosh H, Hershman D (2006). HER-2/neu overexpression in uterine papillary serous cancers and its possible therapeutic implications. Int J Gynecol Cancer.

[24] Todeschini P, Cocco E, Bellone S (2011). Her2/neu extracellular domain shedding in uterine serous carcinoma: implications for immunotherapy with trastuzumab. Br J Cancer.

[25] Togami S, Sasajima Y, Oi T (2012). Clinicopathological and prognostic impact of human epidermal growth factor receptor type 2 (HER2) and hormone receptor expression in uterine papillary serous carcinoma. Cancer Sci.

[26] Buza N, Roque DM, Santin AD (2014). HER2/neu in Endometrial Cancer: A Promising Therapeutic Target With Diagnostic Challenges. Arch Pathol Lab Med.

[27] Ramalingam P, Masand RP, Euscher ED, Malpica A (2016). Undifferentiated Carcinoma of the Endometrium: An Expanded Immunohistochemical Analysis Including PAX-8 and Basal-Like Carcinoma Surrogate Markers. Int J Gynecol Pathol..

[28] English DP, Roque DM, Santin AD (2013). HER2 expression beyond breast cancer: therapeutic implications for gynecologic malignancies. Mol Diagn Ther.

